# The Effectiveness of Cognitive-Existential Group Therapy on Increasing Hope and Decreasing Depression in Women-Treated with Haemodialysis

**DOI:** 10.5539/gjhs.v8n6p219

**Published:** 2015-11-17

**Authors:** Bahman Bahmani, Maryam Motamed Najjar, Mansour Sayyah, Abdollah Shafi-Abadi, Hamed Haddad Kashani

**Affiliations:** 1 Department of Counseling, University of Social Welfare and Rehabilitation Sciences, Tehran, Iran; 2 Department of Counseling, Islamic Azad University-Science and Research Branch, Tehran, Iran; 3 Trauma Research Center of Kashan University of Medical sciences, Kashan, Iran; 4 Department of Counseling, University of Allameh Tabatabaii, Tehran, Iran; 5 Anatomical Sciences Research Center, Kashan University of Medical Sciences, Kashan, Iran

**Keywords:** cognitive-existential treatment, hopefulness, depression, hemodialysis, female

## Abstract

**Introduction:**

Hopefulness is one of the most significant predictors of adaptation in hemodialysis patients, and plays a vital role in the recovery process. In contrast to hopefulness, depression is a frequent psychological reaction of the hemodialysis treatment with many negative consequences. The current research was designed to examine the effect of cognitive-existential treatment on the level of hopefulness and depression in hemodialysis patients.

**Materials & Methods:**

This quasi-experimental research included 22 female patients suffering from chronic kidney failure disease undergoing hemodialysis treatment for at least 3 months. The patients were randomly assigned into two groups of experimental and control conditions. The experimental group received a combination of treatment including some elements of “existentialism” philosophy and a “cognitive” approach designed for the Iranian population. The treatment protocol lasted for 12 sessions of 90 minutes twice per week prior to the entry of the patient to the dialysis session. Miller’s hope scale and BDI-II-21 were employed to collect the data. Statistical analysis was performed on the data using analysis of covariance by SPSS: 16 software.

**Results:**

The result of the analysis indicated that there was a significant improvement in hopefulness level and decrease in depression of the patients in the experiment condition (P<0.01).

**Conclusion:**

The result of analysis showed that cognitive-existential treatment resulted in the increase of hopefulness and decrease level of depression in the hemodialysis patients suffering from chronic kidney failure.

## 1. Introduction

The prevalence and incidence of kidney failure treated by dialysis and transplantation in the United States have increased from 1988 to 2004 (Josef Coresh et al., 2007). A preliminary estimate suggests that by 2011 about 40 thousand people are whom chronic kidney disease existed in Iran (Larijani, 2004) throughout the world, and approximately 90% of patients with end-stage renal disease (ESRD) begin renal replacement therapy with hemodialysis (Kimmel & Peterson, 2005). The prevalence of renal failure in a world is about of 242 million people and this amount is increased annually by about 8% (Brunner, 2009). Hemodialysis is a lifelong treatment that frequently leads to adverse effects on patients’ mental health (Kimmel, 2001). Depression is the most common psychological complication which has serious impact on the quality of life of hemodialysis patients and their caregivers, affecting negatively their social, economic and psychological well-being.

Considering that the renal disease may seem an end stage disease, it significantly affects the lives of patients. That is, due to the loss of many important functions such as kidney function, family role, work role, sexual function, time and mobility, the disease may lead to the experience of despair (Kimmel et al., 2005), constraint, fear of death, and dependency upon treatment (De-Nour, Shaltiel, & Czaczkes, 1968; De-Nour, 1982; O’Brien, 1990; Reiss, 1990). In turn it is argued that the latter may affect the quality of life, and also exacerbate feelings of a loss of control (Levenson et al., 1991). Due to the poor prognosis of the disease and associated complications it has devastating consequences on physical as well as psychological condition of the patients. Other reported difficulties associated with chronic renal failure is impaired cognitive functioning (Billington et al., 2008) and higher level of anxiety (Kutner & Brogan, 1990) as compared normal Therefore, considering the untreatable condition of renal disease and the very serious consequence of terminating dialysis, it may be concluded that the natural reaction of these patients to the chronic insufficiency of kidney failure is depression (Dogan et al., 2005; Kimmel & Peterson, 2005; Kimmel & Levy, 2001). In fact, different signs such as sorrow, limitation of activity and interest concentration difficulties, difficulty in thinking, considerable increase or decrease in appetite and sleep, helplessness, despair, hopelessness and in some cases suicidal thoughts (and actions) are frequently observed (Billington et al., 2008).

Hedayati and associates found that 26.7% of the patients in hemodialysis treatment center suffered from depression; 65% of the patients were severe depression, 27% were afflicted to moderate depression, and 8% were experiencing mild depression (Hedayati et al., 2008). Meanwhile, there are many research reports that indicate there is an inverse association between the feeling of hope and depression and anxiety (Sherwin et al., 1992; Bejestani, 2009). Hope is one of the most important factors in cognitive adaptation of chronically ill patients. Hope has been considered as a positive construct that has had positive effects on the improvement of hard to treat diseases (Barunum et al., 1998; Craig, 1983; Dubree & Vogelpohl, 1980; Mcgree, 1984). It is listed as one of the strategies to cope with such diseases (Baldreeet al., 1982). Several research results have indicated that high levels of hope are a good indicator for the cooperation of patients with the treatment andit leads to positive mood, healthy mentality, and good body system immunity (Peterson, 2000; Snyder, 2002; Scheier; Carver, 1985).

A clearer definition of hope is given by Snyder (1994, 1995) who defined it a process in which an individual set goals, adopts a strategy to reach the goal, and creates and maintains the motivation to thrive for attaining goals. Despite the fact that several researches have been conducted to examine the role of hope in treatment of hard to treat diseases such as chronic pain (Affleck, 1996), cancer tumors (Elliot et al., 1991), visualimpairment (Jackson et al., 1998), and burn injuries (Barunum at al., 1998). It seems that research regarding the effectiveness of hope in treating the hemodialysis patient is still in its infancy (Billington et al., 2008). Billington and associates have demonstrated that the most significant predictor of adaptation to the various psychological reactions to hemodialysis treatment was “hope”. This variable played a vital role in the process of treatment. Despite the progress in the field of medical treatment and psychological interventions to help the hemodialysis patients, this group of patients has many worries about their life including the fear of death, isolation, loneliness at the beginning of their treatment that is expected, as mentioned negatively affect adaptation to treatment as well as compliance (Billington et al., 2008).

Scientists found that patients who suffer from hard to treat disease experience the fear of death, loss of meaning, grief, loneliness and loss of freedom. This author states that since these patients experience such negative excitement, they may become disappointed and even lose self-respect (Kissane et al., 2003). They considers existential contents such as concerns about death, loss of meaning, grief, loneliness, loss of freedom and loss of worthiness as existential key challenges for the patients with incurable disease, whom the people think they have a high probability of death. In addition, he considers that the patients may, as a result of experiencing negative excitements related to existential contents, suffer from intense losing of spirits or what is termed by him as “existential harm”, and have a feeling of inadequacy (Kissane et al., 2003).

Psychological treatments in this regard have been employed to reduce or alleviate the psychological disturbances associated with the medical treatments. Cognitive behavioral treatment research demonstrated that the level of depression in treatment of hemodialysis patients was less than the control group (Duarte et al., 2009). It can be argued there are many challenges facing the hard to treat patients (Kissane et al., 2003) and carefully designed research using psychological interventions to reduce the despair and depression of these patients is often necessary. One of the interventional approaches to deal with the anxiety in breast cancer patients is the new method that combines two methods of the Beck’s cognitive psychotherapy (1975) and Yalom’s existential psychotherapy (1980). In this method, the patient has the opportunity to express his/her feeling with the present anxieties and admit them while using the cognitive strategies, i.e., identifying, changing and correcting the various cognitive errors that result in keeping certain behaviors intact.

This method is a combination of cognitive re-evaluation (Moorey & Greer, 1989) and improving the coping skills (Fawzy et al., 1990) that is integrated with existence strategic factors (Yalom, 1980) and supportive/instrumentation (Kissane et al., 1997), while attempt has been made to use the existing supportive benefit in groups to form intervention that are used in the framework of group counseling (Bahmani, 2010). Previous research was conducted to compare the method of education oriented cognitive intervention (Furr, 1998) and existential -cognitive treatment method (Kissane et al., 1997) to improve the psychological stress of depressed women suffering from breast cancer and showed that the mean score of hope in the existential-cognitive treatment method improved significantly and the level of their depression decreased.

In this regard, it seems like there is sufficient evidence to show the effectiveness of existential-cognitive treatment method for reducing the level of depression and increase the hope of patients suffering from treatment for dialysis. Therefore, the researcher proposed this research to examine a method that considers the needs of patients under special treatment such as dialysis who need social support, face loneliness, isolation, sad feeling of facing death, losing the opportunity to job, losing education, and emotional difficulties and the pain of treatment method.

Given the importance of both existential and cognitive approaches it may be important to discuss them in more depth- what they focus on, and how their related to the experience of depression and the like.

## 2. Methods and Materials

This was a quasi-experimental research in which all the female patients referred to Hashemie nejad Hospital for dialysis treatment were randomly assigned into two equal groups (n=11). The patient were all suffering from chronic kidney failure and were required to refer to the treatment center 2 to 3 times per week. In order to match the demographic characteristics of the patients, an interview session was arranged and information such as age, education level duration of treatment (at least 4 month), and medication history was recorded, and finally 22 patients with the inclusion criterion were selected.

In a preliminary session, the patient were asked to complete the Beck Depression Inventory II (BDI-II) and Miller Hope Scale (MHS). Following the explanation of the procedures and the completion of consent the patients were randomly divided into two groups of experimental and control. The treatment included 12 sessions of 90 minutes of two day per week consultation prior to the dialysis room. Two of the participants in the experimental group withdrew their participation due to personal problems.

The instrument employed in this research was as follows:

1) Personal and demographic data: in this form, information such as age, level of education, ethnicity, cause of disease, duration of treatment by dialysis, medication.

2) Miller Hope Scale (MHS): the hope scale (Miller & Powers, 1988) includes 48 aspects of behavior that assesses hope or despair based on the overt or covert demonstration of behavior. The score ranges from 40 to 200(40 defines complete despair and 200 as the maximum of hope). Internal consistency of this scale has been reported by Salimi Bejestani (2010) with Cronbach alpha equal to 0.92.

The Beck Depression Inventory-II; this questionnaire was designed in 1996 after the revision of the original version. This form includes 21 question items that range in response from 0 to 3. The tool is used for self-report of signs of depression for individuals above 13 years old and higher. The questionnaire reveals the presence and the severity of depression on a four-choice option scale. The score lower than 14 defines the minimum level of depression, between 14 to 19 is considered mild, 20 to 28 is moderate, and 29 to 63 is interpreted as a high level of depression. Dobson and Mohammad Khani (2007) examined the total reliability (r=0.91) of the test and the correlation coefficient between the subscale and the total score (question discrimination power) between 0.454 to 0.681. The convergent validity of this scale was tested by Bahmani (2010) by calculating the correlation coefficient between the scale and brief symptom inventory (BSI).

## 3. Results

Analysis of covariance was used to test the significant mean difference. This method can statistically omit effects of pre-test on post- test and then analysis differences which observed between means of comparative groups. Results of ANCOVA presented in Table 1.

**Table 1:** Mean, SD and ANCOVA of hope and depression

Variables	Control group				Experimental group				ANCOVA
	
	Post test		Pre test		Post test		Pre test		
				
	SD	M	SD	M	SD	M	SD	M	F
Hope	27.72	150.09	28.29	162	33.91	189.37	21.41	164.75	23.89***
Depression	10.46	18.54	9.01	19.09	8.77	8.75	9.73	16.37	38**

*** P< 0.001.

** P< 0.01.

As results shown in the above table, the mean of scores acquired by member of experimental group in post-test for hope is significantly higher (164.7<189.37) and for depression is lower (8.75<16.37) than pre-test. While the values of hope and depression for control group did not change significantly. Therefore it can be concluded that in this study cognitive existential group therapy as independent variable could increase hope and decrease depression as dependent variables.

## 4. Discussion

The result of this research indicated that cognitive-existential group therapy significantly changed the level of depression and hope of the patient women who were treated by dialysis. While the treatment increased the level of hope, it decreased the level of depression in this group of patients. Such an improvement may be attributed to the multidimensionality of the treatment program. Each component of the program might have led to this desirable change in hope and depression condition. The first component is the presence in the group.

It seems, attendance in the group positively changed their hope and depression. Yalom (2001) claimed that the most of patients are facing a conditions that is life threatening, that leads to the painful experience and conditions that eventually result in suppression of feelings and they gradually become more stranger to their “self-existence”.

Furthermore, the majority of patients believe that their problem is unique and do not have long time lived their life. The presence in the group provides the opportunity for the members to know each other and learn that in some instances there are people who are in worse condition than they are and in some instances they are in better condition than the others (Gorey, 2010). This is a curative characteristic of therapeutic groups which yalom called universality experience. The individual finds the chance to learn about the life experiences of other patients who have had the patience, tolerance, efforts and success or failure. All these times and presences provides the unique opportunities for the patient to interact with others; thus helps them to overcome the feeling of helplessness, isolations, miseries, apathies, being discriminated against, dark destiny and live with a more positive and realistic feeling toward the life (Yalom, 1980).

The second component in the confirmation of the hypothesis is the cognitive-existential approach.

Kissane and associates (1997, 2002) and Bahmani (2010) believe that in individuals, who are involved in chronic disease, the meaning and hope will diminish over time and the feeling of depression is intensified. Therefore, existential therapies with a combination of cognitive strategies are the most suitable treatment methods for reducing depression and raising hope among this group of patients.

In the cognitive approach, change in emotions depends on change in cognition and beliefs of individuals. Cognitive therapists attempt to identify etymology of false patterns and irrational cognition in the past and how it continued up to the present time and try to teach the patient how to identify these dysfunctional thoughts and beliefs to change (Bahmani, 2010). Kissane and associates (1997) believes that the group can take important steps to help the individual by adopting courageous intervening methods that straightly deal with the fears and worries of the patient at the same time that try to correct the inappropriate and illogical cognitive patterns. Combining the cognitive and existential method in group therapy succeeded in playing a significant role in reducing depression and increase hope in patients with chronic diseases; an expectation that the result of this study also confirmed it.

Snyder (2000) considers hope as a mean by which the patient may rely on to reach desirable goals despite the presence of difficult conditions. In addition, the author considers the presence of motivation as a factor that facilitates the path to reach the goals. This author also considers “hope therapy” as a construct similar to what Kissane (1997) views cognitive-existential therapy from this prospect. The cognitive-existential group therapy method is a combination of cognitive re-evaluation technique (Moorey & Greer, 1989), improving coping skills (Fawzy et al., 1990) and is combined with factors from existential strategies (Yalom, 1980) and supportive instrumental strategies (Kissane et al., 1997) and in the same time in order to gain the supportive benefits of group meetings, the intervention performed in group counseling format (Bahmani, 2010).

Therefore, it was expected that the cognitive components of intervention in this research result in decreasing the distortive cognitive components of thoughts such as empathy and loneliness of the patients as was shown by the increase in hope that was observed in the results of this study. The increase in hope in this research was also in agreement with the result of research reported by Bahmani (2010), Kimmel (2000), and Scheier et al. (2000). Due to the lack of data regarding the interventions that have been introduced for increasing the hope in hemodialysis patients, no definite research result is available in the literature.

## 5. Conclusion

The researcher suggests that the effectiveness of applying cognitive-existential therapy for the dialysis patient need to be compared with the other types of therapies such as cognitive behavior, existential, instrumental emotional group therapy. In addition, the effectiveness of this therapy method to treat for hemodialysis patients’ needs to be compared with its effectiveness in treating the men in hemodialysis treatment.
